# Phase-dependent dynamics of circulating cell-free mitochondrial DNA reflect distinct immunometabolic states

**DOI:** 10.1590/S1678-9946202668048

**Published:** 2026-07-24

**Authors:** Karen Alessandra Rodrigues, Emilly Henrique dos Santos, Gabriel Acca Barreira, Mariana Okay Saippa, Maria Carolina Pires Cruz, Kelly Aparecida Kanunfre, Thelma Suely Okay

**Affiliations:** 1 Universidade de São Paulo, Faculdade de Medicina, Instituto de Medicina Tropical de São Paulo, Laboratório de Soroepidemiologia, São Paulo, São Paulo, Brazil; 2 Universidade de São Paulo, Faculdade de Medicina, Hospital das Clínicas, Laboratório de Pediatria Clínica (LIM-36), São Paulo, São Paulo, Brazil; 3 Universidade de São Paulo, Faculdade de Medicina, Departamento de Pediatria, São Paulo, São Paulo, Brazil; 4 aculdade Israelita de Ciências da Saúde Albert Einstein, São Paulo, São Paulo, Brazil; 5 Faculdade de Medicina do ABC, anto André, São Paulo, Brazil; 6 Universidade de São Paulo, Faculdade de Medicina, Hospital das Clínicas, Laboratório de Investigação Médica em Imunologia (LIM-48), São Paulo, São Paulo, Brazil

**Keywords:** Cell-free mitochondrial DNA, qPCR, tuberculosis, Chronic pediatric diseases, Systemic inflammation, Biomarker

## Abstract

Circulating cell-free mitochondrial DNA (ccf-mtDNA) has emerged as a potential biomarker of tissue injury and systemic inflammation, acting as a mitochondrial damage-associated molecular pattern (DAMP) capable of activating innate immune pathways. However, most studies have focused on acute inflammatory conditions, and the behavior of ccf-mtDNA across distinct immunological and metabolic disease states remains poorly characterized, particularly in pediatric populations. This study investigated whether circulating ccf-mtDNA levels vary according to the clinical and immunometabolic context rather than simply reflecting the presence of inflammation. Serum ccf-mtDNA copy number was quantified by quantitative polymerase chain reaction (qPCR) targeting the mitochondrial ND2 gene in 181 clinical samples obtained from five groups: adults with chronic-active and/or treatment-refractory pulmonary tuberculosis (n = 47); asymptomatic children with latent tuberculosis infection confirmed by interferon-gamma release assay (IGRA) without clinical, radiological or microbiological evidence of active disease (n = 11); children with severe chronic underlying diseases in clinically stable condition (n = 41); children undergoing cardiac surgery with cardiopulmonary bypass (CPB), with perioperative serial sampling (n = 52); and healthy young adult blood donors as controls (n = 30). Non-parametric statistical tests were applied due to non normal data distribution. Median ccf-mtDNA levels in controls were 649.3 copies/μL. Adults with chronic-active pulmonary and/or treatment-refractory tuberculosis and children undergoing cardiac surgery with CPB did not exhibit significantly elevated ccf-mtDNA levels compared with controls. In contrast, significantly higher levels were observed in IGRA-positive children with latent tuberculosis infection (median: 1,648.5 copies/μL; p = 0.0004) and in children with severe chronic underlying diseases despite the absence of overt infection or inflammation (median: 2,663.9 copies/μL; p = 0.0001). These findings suggest that circulating ccf-mtDNA does not behave as a simple linear marker of inflammatory intensity. Instead, its levels appear to reflect the interaction between mitochondrial injury, immune activation, metabolic competence, and clearance mechanisms. We propose a phase-dependent model in which mitochondrial DAMP signaling is amplified during sustained but metabolically competent immune engagement, and is attenuated during advanced immunometabolic exhaustion. These findings suggest that ccf-mtDNA levels are shaped by the host's underlying immunometabolic context, rather than simply mirroring the intensity of systemic inflammation.

## INTRODUCTION

Mitochondria are central regulators of cellular metabolism, survival, and immune signaling. In addition to their essential role in ATP production through oxidative phosphorylation, mitochondria integrate bioenergetic demands with innate immune responses and inflammatory signaling pathways. During infection, tissue injury, or systemic inflammatory stress, immune cell activation is frequently accompanied by mitochondrial dysfunction, oxidative stress, and activation of intrinsic apoptotic pathways^1,2^. Excessive mitochondrial injury and immune-cell apoptosis have been implicated in a range of pathological conditions, including sepsis and critical illness, where they contribute to immune dysregulation and organ dysfunction^3,4^.

Beyond their metabolic functions, mitochondria also serve as important mediators of innate immune activation. Human mitochondrial DNA (mtDNA) is a circular double-stranded molecule of approximately 16.6 kb encoding 37 genes essential for oxidative phosphorylation^5^. Due to the evolutionary origin of mitochondria from ancestral α-proteobacteria, mtDNA retains bacterial features, including unmethylated CpG motifs^6^. When mitochondrial integrity is compromised, mtDNA can be released from mitochondria into the cytosol and subsequently into the extracellular compartment as circulating cell-free mitochondrial DNA (ccf-mtDNA). In the extracellular environment, mtDNA functions as a damage-associated molecular pattern (DAMP) capable of activating innate immune pathways^6,7^.

Experimental and clinical studies have demonstrated that extracellular mtDNA can activate multiple inflammatory signaling pathways. Internalized mtDNA can stimulate Toll-like receptor 9 (TLR9) in endosomal compartments^6,8^ while cytosolic mtDNA activates the cyclic GMP-AMP synthase-stimulator of interferon genes (cGAS-STING) pathway, leading to transcription of type I interferons and pro-inflammatory cytokines such as interleukin-6^9,10^. In addition, mitochondrial dysfunction and oxidized mtDNA contribute to activation of the NLRP3 inflammasome, further amplifying inflammatory responses^11,12^. Through these mechanisms, mitochondrial DNA represents an important interface between metabolic stress and innate immune activation^13^.

Consistent with these biological properties, elevated circulating ccf-mtDNA levels have been reported in several acute inflammatory conditions, including trauma, sepsis, and systemic inflammatory response syndrome^7,14^. In these settings, circulating mtDNA is commonly interpreted as a marker of tissue injury and systemic hyperinflammation, and higher levels have been associated with disease severity and increased mortality in critically ill patients^7,15^. However, most available studies have focused on acute, high-intensity inflammatory states.

Whether circulating ccf-mtDNA behaves similarly across a broader spectrum of clinical conditions remains uncertain. In particular, little is known about its behavior in chronic or latent infections, as well as in clinically stable patients with underlying chronic diseases characterized by persistent immune dysregulation or metabolic stress. These contexts may differ substantially from acute inflammatory syndromes in terms of immune activation, mitochondrial turnover, and metabolic reserve.

Chronic infectious diseases such as pulmonary tuberculosis represent a model of prolonged antigen exposure and sustained host-pathogen interaction. Progressive disease has been associated with T-cell exhaustion and impaired immune responsiveness^16,17^. At the opposite end of the spectrum, latent tuberculosis infection (LTBI), identified by interferon-gamma release assays (IGRAs), is traditionally considered clinically silent but is increasingly recognized as an immunologically dynamic state characterized by persistent immune surveillance and host-pathogen equilibrium^18,19^.

Non-infectious conditions can also contribute to chronic mitochondrial stress. Children with chronic and severe underlying diseases including inborn errors of immunity, malignancies, organ failure, or post-transplant states, frequently experience chronic oxidative stress and immune dysregulation^20,21^. In contrast, cardiac surgery with cardiopulmonary bypass (CPB) represents a controlled model of acute systemic inflammatory stress driven by ischemia-reperfusion injury and extracorporeal circulation^22^.

Despite growing interest in mitochondrial DAMPs as biomarkers of inflammation, comparative analyses across these diverse clinical contexts remain scarce, particularly in pediatric populations. Importantly, it remains unclear whether circulating ccf-mtDNA functions as a universal marker of inflammatory severity or whether its levels instead reflect distinct immunometabolic phases of disease.

In the present study, circulating ccf-mtDNA copy number was quantified in serum samples using quantitative polymerase chain reaction (qPCR) targeting the mito­chondrial ND2 gene, a relatively stable and highly variable region of the mitochondrial genome that allows reliable species identification and mtDNA copy number estimation^5^. We hypothesized that circulating ccf-mtDNA levels would not simply correlate with the presence of inflammation or infection but would vary according to the immunometabolic phase of disease. Specifically, we postulated that ccf-mtDNA levels would be elevated in conditions characterized by sustained yet metabolically competent immune activation and attenuated in advanced states of immune exhaustion or metabolic compromise.

To test this hypothesis, we measured serum ccf-mtDNA copy number in five groups representing distinct inflammatory and immunological contexts: adults with chronic-active and/or treatment-refractory pulmonary tuberculosis; children with latent tuberculosis infection; pediatric patients with severe chronic underlying diseases; children undergoing cardiac surgery with cardiopulmonary bypass; and healthy young controls. By comparing these distinct clinical conditions, we aimed to determine whether circulating ccf-mtDNA represents a universal biomarker of inflammation or rather reflects the dynamic interplay between mitochondrial injury, immune activation, and host metabolic reserve.

### Ethics

This study was approved by the Institutional Ethics Committee of the Hospital das Clinicas, Faculdade de Medicina, Universidade de Sao Paulo (process CAAE Nº 73522723.0.0000.0068) on October 19, 2023. Informed consent was obtained by the attending physician from parents or legal guardians, and informed assent was obtained from school-aged children and adolescents.

## MATERIALS AND METHODS

### Participants

The study included four patient groups and one control group, totaling 151 clinical samples and 30 control samples.

### Adult chronic-active pulmonary tuberculosis (TB) disease

Adults with active pulmonary TB were followed at the TB outpatient clinic of HC/FMUSP. Diagnosis was confirmed by sputum smear microscopy (Ziehl-Nielsen staining) and isolation of *Mycobacterium tuberculosis* in cultures (Loweinstein-Jensen medium). Thirty-one patients (10 women, 21 men; 18-69 years) had chronic-active complicated TB due to multiple prior treatment failures, poor adherence, and/or drug resistance. GeneXpert testing was unavailable at that time. Concomitant infections (HIV, syphilis, viral hepatitis) were excluded. These 31 patients had samples collected at the time of diagnosis (TB disease 1st group). Unfortunately, only 16 out of 31 patients had a second blood sample collected after six months of treatment (TB disease 2nd group).

### Pediatric patients with latent TB infection

Eleven asymptomatic children (7 girls and 4 boys), with a median age of 58.5 months and documented epidemiological exposure to tuberculosis, were followed at HC/FMUSP. All participants were asymptomatic, had normal chest radiographs, and C-reactive protein (CRP) levels within the reference range, as confirmed by a pediatric infectious disease specialist who evaluated all of them before recruitment and blood sample collection. Microbiological investigations for TB were negative in all cases, including repeated negative sputum or gastric lavage smear microscopy and negative cultures. All had received prior BCG vaccination, confirmed by their vaccination cards. Latent TB infection was established based on a positive Interferon-Gamma Release Assay (IGRA).

### Pediatric patients with severe chronic underlying diseases

Forty-one pediatric outpatients (21 boys and 20 girls; mean age 47 months) with severe chronic medical conditions were included. These conditions comprised genetic disorders predisposing to recurrent infections, inborn errors of immunity, organ failure or solid organ transplantation, and malignancies. At enrollment, no participant had evidence of acute infection or inflammation, as confirmed by the clinical evaluation carried out by a pediatric infectious disease specialist. Patients with malignancies were not receiving chemotherapy at the time of inclusion. All participants had non-infectious pattern in their complete blood count, C-reactive protein (CRP) levels within the normal range, and negative blood cultures at enrollment.

### Children with congenital heart disease requiring surgical correction

Thirteen children (6 girls, 7 boys; 4-26 months) with large ventricular or atrial septal defects underwent surgical correction with cardiopulmonary bypass (CPB). Preoperative evaluation (total blood count, CRP and blood cultures) showed no evidence of systemic inflammation or infection.

### Control group

The control group consisted of healthy young adults aged 18-24 years. Although not fully age-matched to the younger participants, their inclusion was justified by institutional restrictions that do not have healthy children and adolescents, therefore precluding blood collection from matched controls. According to the World Health Organization^23^, the category of "young people" encompasses individuals aged 10-24 years, including adolescents (10-19 years) and youth (15-24 years). Moreover, pediatric care in many healthcare systems, including those in the United States, commonly extends through 21 years of age. Therefore, the use of healthy young adults provided a practical and conceptually appropriate comparison group within the broader framework of young people.

### Sample collection

In adults with active TB, serum samples were collected at diagnosis (TB disease 1th - n=31) and after six months of treatment (TB disease 2nd - n=16), totaling 47 samples in this chronic-active TB disease group.

Pediatric patients with latent TB infection and those with severe chronic underlying diseases provided one serum sample each for the research, at the time of enrollment (n=11 and n=41, respectively). They also had the following laboratory testes performed: total blood count, CRP and blood cultures.

In children with congenital heart disease, samples were collected in four time points: immediately before CPB, immediately after CPB, 24 and 48 h after CPB. The procedure duration ranged from 1 h and 45 min up to 180 min (3 h). The total number of samples was 52 in this group (13 per time point). These patients also had total blood count, CRP and blood cultures performed before surgery. Control participants provided one serum sample each (n=30).

### Statistical analyses

Data distribution was assessed using the Shapiro-Wilk (n<50) or the Kolmogorov-Smirnov (n>50) test. As variables showed a non-normal distribution, non-parametric tests were applied.

Comparisons between two unpaired groups were performed using the Mann-Whitney U test, while the Wilcoxon's signed-rank test was used to compare paired data from two groups. For comparisons involving more than two groups, the Kruskal-Wallis test was applied for unpaired data or the Friedman test to paired data. When significant differences were detected, the Dunn's post hoc test with p-values corrected by Bonferroni was used to identify pairwise differences.

### Laboratory methods

### Serum processing

Blood was collected in gel-separator tubes, centrifuged (10 min at maximum speed), and serum stored at −20 °C. Before use, aliquots were thawed, centrifuged twice under the same conditions, and the debris-free supernatant stored at −20 °C until DNA extraction.

### Ccf-mtDNA extraction

Cell-free mtDNA was extracted from 200 μL of previous ultracentrifuged serum samples using the QIAamp DNA Mini Kit (QIAGEN) according to the manufacturer's protocol. DNA concentration was measured with the Nanodrop 1,000 spectrophotometer (Thermo Fisher Scientific) and afterwards by the Qubilt (Thermo Fisher Scientific) nucleic acids fluorometer, and DNA concentrations from serum samples extractions fell below the limit of detection regarding both equipments.

### qPCR quantification

qPCR was performed on an ABI StepOne Real-Time PCR System with QuantiFast SYBR Green Master Mix (QIAGEN) using MT-ND2 primers (forward: 5’-CACAGAAGCTGCCATCAAGTA-3’; reverse: 5’-CCGGAGAGTATATTGTTGAAG AG-3’), generating a 90 bp amplicon. Cycling: 95 °C for 10 min; 40 cycles of 95 °C for 15 s and 60 °C for 60 s.

### Cloning and plasmid preparation

Amplicons were purified (ExoSAP-IT™, Applied Biosystems) and cloned into pJET1.2 (CloneJET PCR Cloning Kit, Thermo Fisher Scientific). Competent E. coli were transformed by CaCL_2_ heat shock, and positive colonies were confirmed by plasmid extraction (alkaline lysis). DNA was precipitated, washed, resuspended in sterile water, quantified, and adjusted to 20 ng/μL.

### Standard curve

Plasmid copy number was calculated using the Applied Biosystems formula^24^ to generate a six-points standard curve, each point tested in triplicate.

### Positive control DNA sequencing and validation

Recombinant plasmids were sequenced on an ABI PRISM^®^ 3500 Genetic Analyzer (FMUSP PREMiUM Network) using the BigDye^®^ Terminator Kit. Products were purified (BigDye^®^ XTerminator^™^), electropherograms edited in BioEdit, and sequences aligned with CodonCode Aligner. BLAST analysis confirmed 100% identity with human mitochondrial MT-ND2 (GenBank: positions 4471-5512 bp).

### qPCR standard curve preparation and test of clinical samples

**Figure 1:**
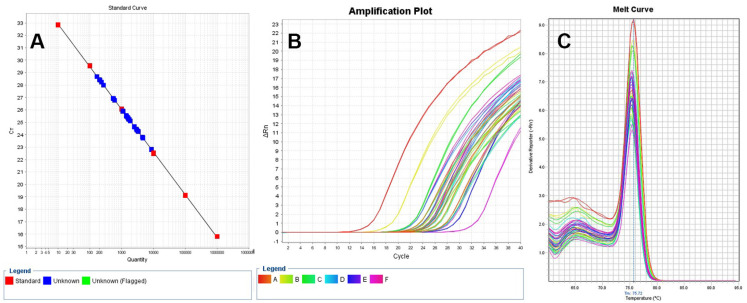
gives an example of a Real Time PCR experiment performed with a six-points standard curve: (A) standard curve points are in red, and clinical samples in blue dots); each point of the standard curve was tested in triplicate (B); and the corresponding melt curve is on plot (C).

A standard curve was generated using serial dilutions (10^6^ to 10^9^ copies) of a cloned 90 bp ND2 fragment used as the qPCR-MT-ND2 positive control. Figure 1 shows the standard curve generated from six serial dilutions of the cloned positive control containing known quantities of the target DNA sequence, ranging from 10^6^ to 10^1^ copies/μL. The six points used to construct the standard curve are shown in red, whereas the clinical samples are shown in blue (A). The standard curve exhibited a slope of −3.429, a coefficient of determination (R^2^) of 1.000, and an amplification efficiency of 95.706%; (B) presents the amplification plot obtained from the six serial dilutions of the cloned positive control, ranging from 10^6^ to 10^1^ mtDNA copies/μL, each tested in triplicate, and (C) shows the melt curve, indicating a melting temperature (Tm) of 75.72 °C.

## RESULTS

In the present study, four study groups and a negative control group were evaluated and their characteristics are described in Table 1.

**Table 1:** Key characteristics of participants belonging to the four study groups and the negative controle one.

Group	Participants (n)	Sex (M/F)	Age (range/ mean)	Samples (n)	Key characteristics
a) Adults with pulmonary TB	47	30/15	18-69 y.	47	TB was diagnosed by smear and/or culture. 1st collection time (1st) at diagnosis (n= 31), and 2nd after six months of treatment (n=16)
b) Children with latent TB	11	4/7	Mean 58.5 mo.	11	Latent TB diagnosed by IGRA without signs or symptoms, radiological or microbiological evidence of TB.
c) Children with severe chronic multisystem underlying diseases	41	21/20	Mean 47 mo.	41	Severe chronic diseases (CNS, organ failure/transplant, autoimmune, or neoplasms not under chemotherapy). Patients were stable at sampling, with inaltered total blood count, negative blood cultures and non-increased CRP, and no acute infection or inflammation according to physical examination.
d) Children with congenital heart disease undergoing surgery with CPB	13	7/6	4 - 26 mo.	52	Cardiac surgery with CPB (SIRS model). Samples collected at four time points: immediately before CPB, after CPB, 24 h and 48 h after CPB (13 samples each).
e) Healthy controls	30	15/15	18-24 y.	30	Healthy blood donors.

TB = tuberculosis; IGRA = interferon-gamma release assay; CNS = central nervous system; CPB = cardiopulmonary bypass; SIRS = systemic inflammatory response syndrome; mo.= months; y.= years.

To begin the statistical analysis, normality tests were applied and data distribution was mostly not normal, so that non-parametric tests were applied.

**Figure 2:**
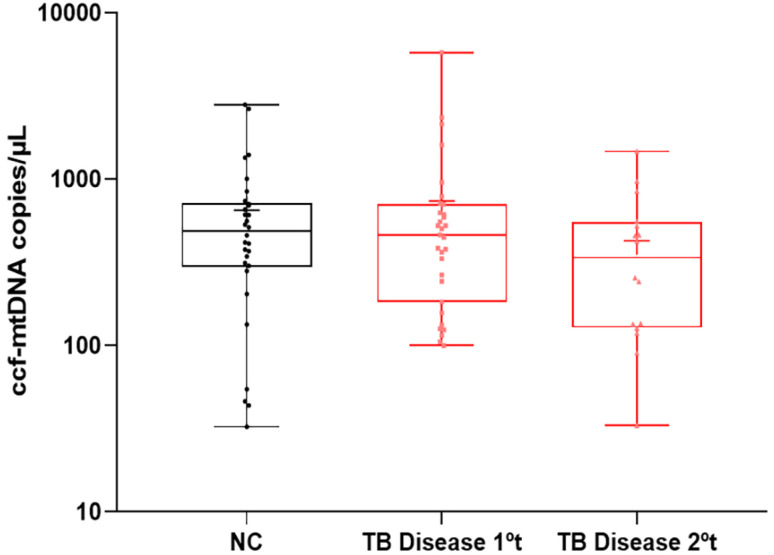
shows the comparison between samples collected from TB disease patients at the time of diagnosis (1st) and after six months of treatment (2nd) with respect to the negative control group. Chronic-active pulmonary TB (median value of ccf-mtDNA in the TB disease 1st is 462.0 copies/μL) compared with the TB disease 2nd results (median of ccf-mtDNA is 337.4 copies/μL), and median of ccf-mtDNA of the negative control group is 486.8 copies/μL). NC = negative control group; TB = tuberculosis; 1st = samples collected at the time of diagnosis; 2nd = samples collected after six months of treatment.

The group of patients with chronic-active treatment-refractory pulmonary TB disease had samples collected at the time of diagnosis (n=31) and after six months of treatment (n=16). These two groups (first collection time – 1st and second collection time – 2nd) and the negative control group (NC) were compared by the Kruskal-Wallis test for multiple comparisons of unpaired samples (Figure 2), resulting in a p-value = 0.4303 (non significant).

**Figure 3:**
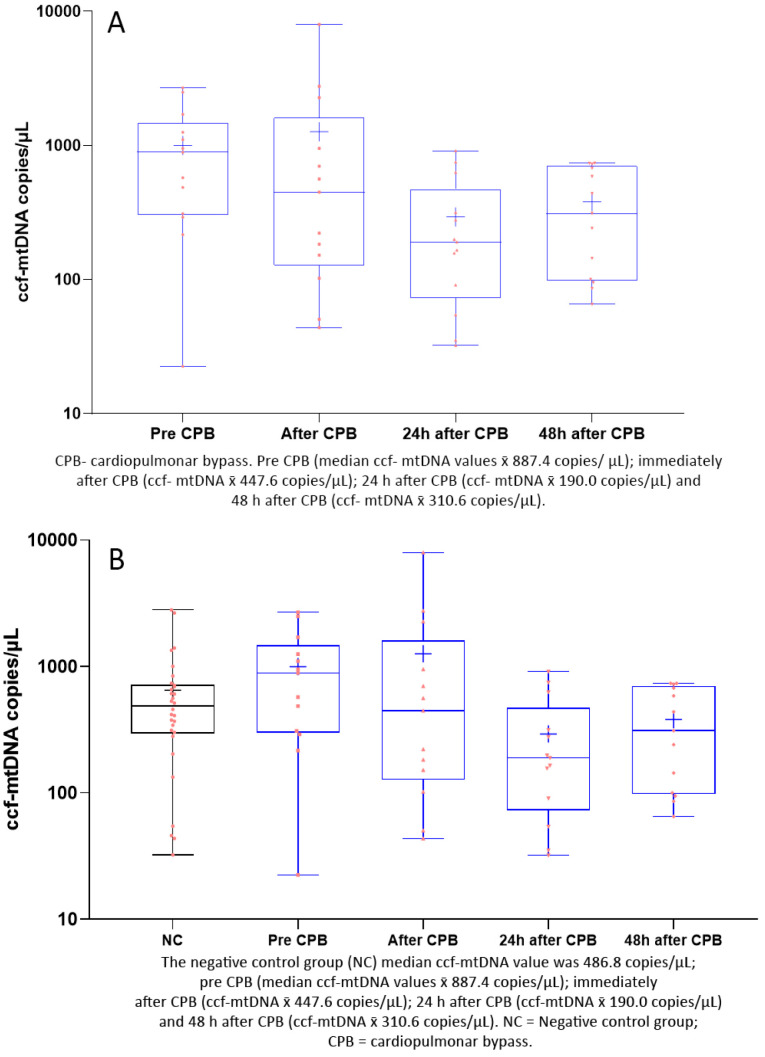
(A) illustrates the Friedman test applied to the 13 patients who underwent cardiac surgery with CPB, sampled four times (immediately before CPB installation; immediately after CPB conclusion; 24 h and 48 h after CPB conclusion); (B) shows the same four time points of patients submitted to cardiac surgery with CPB compared with ne negative control (NC) group by means of the Kruskal-Wallis test for multiple comparisons (unpaired samples).

The next comparison was performed by the Friedman test (three or more groups of related samples – repeated measurements on the same subjects) in the group of children who underwent cardiac surgery with CPB (Figure 3A). There were 13 samples in each of the four time points (pre-CPB, post-CPB, 24 h and 48 h after CPB). The resulting p-value was p= 0.0287 (statistically significant).

As the Friedman test resulted in a statistically significant difference (p=0.0287) (Figure 3A), the Dunn's post hoc test was applied with p-values corrected by Bonferroni, resulting in a slightly significant difference (p=0.0471) in the comparison between pre CPB values vs. 24 h after CPB ones.

Then, we applied the Kruskal-Wallis test (Figure 3B) to compare the four collection times associated with CPB and the negative control (NC) group These multiple comparisons yielded a p-value of 0.0744 (non significant).

**Figure 4:**
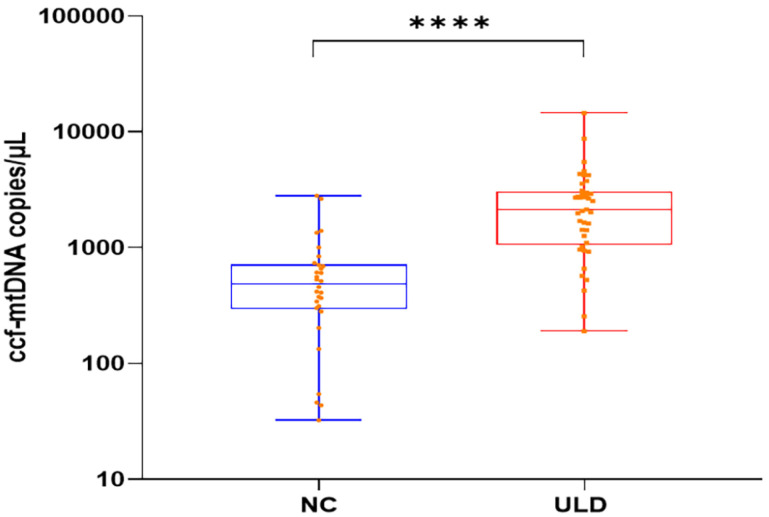
shows the comparison between the negative control group (NC) and the one of pediatric participants with underlying chronic and severe diseases (ULD). The Mann-Whitney test was applied to this pairwise comparison (ccf-mtDNA of NC median 649.3 copies/μL vs. participants with ULD median ccf-mtDNA 2,664.0 copies/μL).

The following procedure was applied to compare the negative control group (NC) and the group of pediatric participants with severe chronic multisystem underlying diaseses (ULD) by using the Mann-Whitney test for unpaired samples (Figure 4), which resulted in a highly significant p-value of 0.0001 (**** in the figure).

**Figure 5:**
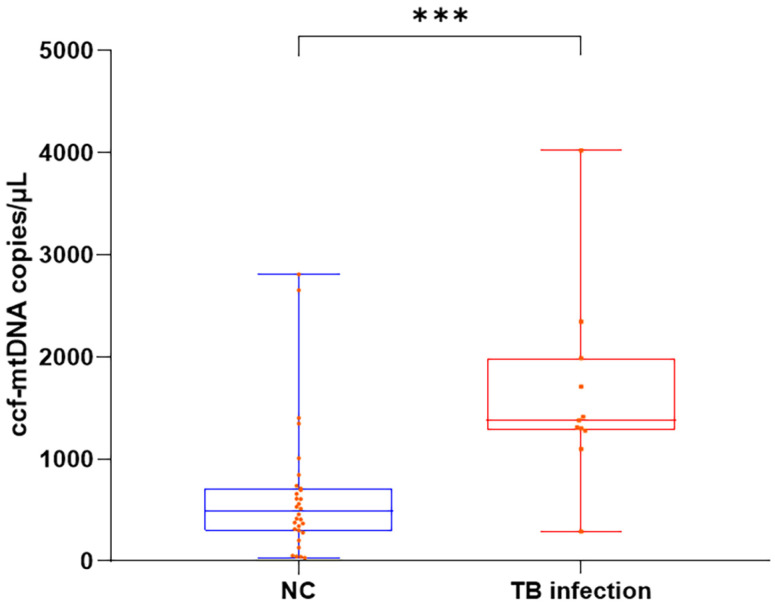
shows the comparsion between the negative control group (NC) and the one of pediatric participants with TB infection. The Mann-Whitney test was applied to the comparison between the negative control group (NC) and the TB infection one (median ccf-mtDNA 649.3 copies/μL of NC $\overset{-}{x}$ median ccf-mtDNA 1.649,0 copies/μL of TB infection).

Lastly, we compared the negative control group (NC) and the pediatric participants with latent TB infection (Figure 5) by using the Mann-Whitney test, which also resulted in a statistically significant difference (p= 0.0004 or *** in the figure).

In summary, the highest ccf-mtDNA levels occurred in children with severe chronic multisystem underlying diseases followed by children with latent TB infection, both groups showing significantly increased ccf-mtDNA levels with respect to negative controls, while the adult patients with chronic-active treatment-refractory pulmonary TB, and children submitted to cardiac surgery with CPB showed no elevation of ccf-mtDNA levels relative to healthy controls.

## DISCUSSION

Circulating cell-free mitochondrial DNA (ccf-mtDNA) has been proposed as an endogenous danger signal released during cellular stress, infection, and tissue injury. Because mitochondrial DNA retains bacterial ancestry and contains unmethylated CpG motifs, it can activate innate immune receptors including Toll-like receptor 9 and cytosolic DNA-sensing pathways such as cGAS-STING, thereby promoting inflammatory signaling and cytokine production^8-12^. Most previous studies have described increased circulating mtDNA levels in acute inflammatory states such as trauma, sepsis, and early active tuberculosis, supporting its interpretation as a marker of tissue injury and systemic inflammation^14,15,18,19^.

The present study revealed a more complex pattern of ccf-mtDNA dynamics across distinct clinical conditions. Rather than correlating directly with the presence of infection or inflammatory stress, circulating ccf-mtDNA levels appeared to vary according to the underlying immunometabolic context.

Unexpectedly, adults with chronic-active treatment-refractory pulmonary tuberculosis did not exhibit elevated circulating ccf-mtDNA levels compared with healthy controls, as previously described^25^. These patients had prolonged disease duration and severe undernutrition, possibly suggesting a state of immune hyporesponsiveness and metabolic compromise rather than active inflammatory hyperactivation. Chronic antigen exposure in advanced tuberculosis has been associated with T-cell dysfunction and exhaustion phenotypes characterized by impaired cytokine production and reduced immune responsiveness^26-28^. Severe malnutrition, frequently present in advanced tuberculosis, further impairs mitochondrial biogenesis and cellular metabolic capacity, potentially limiting mitochondrial stress responses and systemic release of mitochondrial DAMPs^27^. In addition, mitochondrial DNA has been implicated as a mediator of immune paralysis in prolonged inflammatory states^29,30^. Together, these mechanisms may explain why circulating ccf-mtDNA levels remained low despite ongoing infection, suggesting that advanced tuberculosis may represent a state of immunometabolic exhaustion rather than active inflammatory signaling.

A similar pattern was observed in children undergoing cardiac surgery with cardiopulmonary bypass (CPB), in whom circulating ccf-mtDNA levels did not increase significantly across perioperative sampling points. CPB is traditionally associated with systemic inflammatory responses driven by ischemia-reperfusion injury and extracorporeal circulation^31^. However, the magnitude and duration of this inflammatory response are influenced by several factors, including perioperative corticosteroid administration, antimicrobial prophylaxis, and improvements in surgical techniques that reduced CPB duration. In addition, circulating mitochondrial DNA released during acute tissue injury may be rapidly degraded by extracellular nucleases or cleared by phagocytic mechanisms, limiting its detectability in peripheral blood. These factors may collectively contribute to the absence of a measurable increase in circulating ccf-mtDNA in this group^32-36^.

In contrast, elevated ccf-mtDNA levels were observed in children with latent tuberculosis infection. Although LTBI has historically been regarded as a clinically silent condition, increasing evidence indicates that it represents a dynamic host-pathogen equilibrium characterized by persistent antigenic stimulation and ongoing immune surveillance^17,18^. Sustained low-grade mitochondrial stress in immune cells may promote intermittent release of mitochondrial DNA into the cytosol or extracellular space. Once displaced from mitochondrial compartments, mtDNA can activate innate immune pathways through engagement of TLR9 or activation of the cGAS-STING signaling axis. Subthreshold activation of these pathways may promote NF-κB-dependent cytokine production and maintain a state of low-grade inflammatory signaling without overt clinical disease^8-10^

Elevated circulating ccf-mtDNA levels were also detected in children with severe chronic multisystem underlying diseases despite the absence of clinical evidence of acute infection or systemic inflammation. Conditions such as inborn errors of immunity, malignancies, organ failure, and post-transplant states are frequently associated with chronic oxidative stress, mitochondrial dysfunction, and dysregulated apoptosis^19-21^. Persistent cellular stress and mitochondrial turnover in these settings may lead to continuous release of mitochondrial DNA into the circulation. In this context, circulating ccf-mtDNA likely reflects ongoing cellular stress and immune dysregulation rather than pathogen-specific inflammatory processes.

Taken together, these findings indicate that circulating ccf-mtDNA does not behave as a linear marker of inflammatory intensity. Instead, its levels appear to reflect the dynamic balance between mitochondrial injury, immune activation, metabolic reserve, and clearance mechanisms. Conditions characterized by sustained but metabolically competent immune engagement, such as latent infection or chronic systemic stress, may promote detectable release of mitochondrial DNA. In contrast, advanced disease states associated with metabolic exhaustion or immune dysfunction may exhibit reduced mitochondrial danger signaling despite ongoing pathology.

This interpretation supports a phase-dependent model of mitochondrial DAMP dynamics, in which circulating ccf-mtDNA reflects distinct immunometabolic states rather than simple inflammatory burden. Within this framework, mitochondrial DNA release may be amplified during phases of active immune engagement but attenuated during advanced immunometabolic collapse or immune exhaustion.

Several limitations should be considered when interpreting the findings of this investigation. The study groups were heterogeneous and included distinct clinical conditions with potentially different mechanisms governing mitochondrial injury and DNA clearance^5,12^. Sample sizes were limited in some groups, which may have reduced statistical power to detect subtle differences. Circulating mitochondrial DNA levels are also known to exhibit substantial inter-individual variability, influenced by cellular turnover, mitochondrial stress, and nuclease-mediated clearance mechanisms^6^. In addition, strictly age- and sex-matched control groups were not available for each clinical category, reflecting the characteristics of our institution as a tertiary pediatric referral center where most patients present with underlying chronic diseases. In a recently published study^37^, our research team has demonstrated that pre-adolescents, adolescents and young adults with COVID-19 had significantly higher levels of circulating cell-free mtDNA in comparison with a control group composed of young adults (18-incomplete 25 years old) who were blood bank donors. This last control group is similar to the one employed in the present study.

### Limitations

Despite these limitations, the inclusion of diverse clinical contexts allowed exploration of circulating mitochondrial DNA dynamics across a wide spectrum of inflammatory and immunometabolic states. Our findings suggest that mitochondrial DAMP signaling may provide insights into host metabolic and immune competence rather than serving solely as a marker of inflammatory severity.

## CONCLUSION

Our findings indicate that circulating cell-free mitochondrial DNA (ccf-mtDNA) should not be interpreted as a linear surrogate of inflammatory intensity, but rather as a dynamic marker of mitochondrial danger signaling embedded within the host immunometabolic landscape.

Together, these data support the existence of a phase-dependent model in which mitochondrial DAMP signaling is amplified during persistent but functional immune engagement and attenuated during advanced immunometabolic exhaustion. This framework challenges conventional paradigms and highlights the need to interpret ccf-mtDNA within the broader context of mitochondrial integrity, immune competence, and metabolic reserve.

Future studies involving larger, well-matched cohorts and controls and longitudinal sampling will be required to define the temporal dynamics of circulating cell-free mitochondrial DNA and to determine its potential clinical utility as a biomarker for monitoring inflammatory and immunometabolic states in pediatric diseases.

## Data Availability

The anonymized dataset generated during this study is available from the corresponding author upon reasonable request.
